# SWATH Differential Abundance Proteomics and Cellular Assays Show In Vitro Anticancer Activity of Arachidonic Acid- and Docosahexaenoic Acid-Based Monoacylglycerols in HT-29 Colorectal Cancer Cells

**DOI:** 10.3390/nu11122984

**Published:** 2019-12-06

**Authors:** María José González-Fernández, Dmitri Fabrikov, Rebeca P. Ramos-Bueno, José Luis Guil-Guerrero, Ignacio Ortea

**Affiliations:** 1 Food Technology Division, Agrifood Campus of International Excellence, ceiA3, University of Almería, 40120 Almería, Spain; mjgon2@hotmail.com (M.J.G.-F.); dimitri.fabrikov@gmail.com (D.F.); rebeca_ramos_b@hotmail.com (R.P.R.-B.); jlguil@ual.es (J.L.G.-G.); 2 Proteomics Unit, IMIBIC, Reina Sofía University Hospital, University of Córdoba, 14004 Córdoba, Spain

**Keywords:** colorectal cancer, proteomics, SWATH, docosahexaenoic acid, arachidonic acid, HT-29 cells, monoacylglycerols

## Abstract

Colorectal cancer (CRC) is one of the most common and mortal types of cancer. There is increasing evidence that some polyunsaturated fatty acids (PUFAs) exercise specific inhibitory actions on cancer cells through different mechanisms, as a previous study on CRC cells demonstrated for two very long-chain PUFA. These were docosahexaenoic acid (DHA, 22:6*n*3) and arachidonic acid (ARA, 20:4*n*6) in the free fatty acid (FFA) form. In this work, similar design and technology have been used to investigate the actions of both DHA and ARA as monoacylglycerol (MAG) molecules, and results have been compared with those obtained using the corresponding FFA. Cell assays revealed that ARA- and DHA-MAG exercised dose- and time-dependent antiproliferative actions, with DHA-MAG acting on cancer cells more efficiently than ARA-MAG. Sequential window acquisition of all theoretical mass spectra (SWATH)—mass spectrometry massive quantitative proteomics, validated by parallel reaction monitoring and followed by pathway analysis, revealed that DHA-MAG had a massive effect in the proteasome complex, while the ARA-MAG main effect was related to DNA replication. Prostaglandin synthesis also resulted as inhibited by DHA-MAG. Results clearly demonstrated the ability of both ARA- and DHA-MAG to induce cell death in colon cancer cells, which suggests a direct relationship between chemical structure and antitumoral actions.

## 1. Introduction

The fat content of a normal diet consists mainly of triacylglycerols (TAG) (about 90% of total ingested lipids) and small amounts of sterols and phospholipid esters, as well as fat-soluble vitamins (A, D, E, and K) [1]. The fatty acid (FA) distribution on the glycerol backbone of TAG influences their absorption, distribution, and tissue uptake [2]. Free FA (FFA) and *sn*-2-monoacylglycerol (*sn*-2 MAG), the two hydrolysis products of dietary TAG, are absorbed from the lumen into polarized enterocytes in the small intestine. Polyunsaturated FAs (PUFAs) are better absorbed when they are esterified at the *sn*-2 position of the glycerol molecule, while the type of FA at the remaining locations also influences their intestinal absorption [2,3,4,5].

Arachidonic acid (ARA, 20:4*n*6) is a long-chain PUFA (LCPUFA) belonging to the *n*-6 family. This molecule is the precursor of the biosynthetic pathway leading to the production of prostaglandin PGE2, thromboxanes and other metabolites involved in the regulation of various physiological processes, e.g., inflammation and immune function [6]. Docosahexaenoic acid (DHA, 22:6*n*3) is one of the predominant LCPUFAs in the structural phospholipids of the human brain and retina, and it accumulates within the fetal neural tissue during pregnancy and the postnatal period [7]. At present, fish oil constitutes the main source of DHA, although alternatively it can be industrially obtained from several microorganisms, mainly from marine origin, e.g., the unicellular microalga *Crypthecodinium cohnii*, which contains large amounts of DHA [8].

The *n*-6/*n*-3 ratio is commonly used as an index to evaluate the nutritional quality of dietary FA, and it has particular relevance on human health. *n*-3 LCPUFAs help to reduce inflammation, while most *n*-6 LCPUFAs tend to increase it. An imbalanced *n-6/n*-3 ratio contributes to the development of diseases, while an adequate balance helps to maintain and even improve health [9,10].

Different mechanisms have been proposed for the anticancer actions of the various LCPUFAs, including suppression of neoplastic transformation, inhibition of cell proliferation and enhancement of apoptosis [11]. This last phenomenon is morphologically characterized by a decrease in cell and nuclear volume, chromatin condensation and DNA fragmentation, and the presence of lipid bodies, without changes in organelle integrity [12]. Morin et al. [13,14] demonstrated a reduction in cell growth and the induction of apoptosis caused by DHA-MAG, eicosapentaenoic acid (EPA, 20:5*n*3)-MAG, and docosapentaenoic acid (DPA, 22:5*n*3)-MAG, in colon and lung cancer cell lines. In addition, MAG derived from monounsaturated FAs (MUFAs) such as oleic acid (OA, 18:1*n*9), and saturated FAs (SFAs) such as palmitic acid (PA, 16:0), have been related to the decrease in the activity of MRP2 (protein 2 associated with multidrug resistance) in Caco-2 cells at low concentrations [15]. Also, Philippoussis et al. [3] showed that murine T-cells undergo a rapid apoptosis following treatment with different MAG types.

One of the aspects to be considered in the performance of MAG as a cancer inhibitor is the mechanisms for entering into cells. Whereas Schultess et al. [16] stated that MAGs are absorbed by passive diffusion, Ho and Storch [5] suggested that they require a protein-mediated process. Little is known about these input mechanisms, although different enzymes related to biosynthesis and lipid degradation are supposed to be involved in this process. For instance, Acyl-CoA synthetases (ACS) play a critical role in the transport of FA into cells by making this transport unidirectional [17]. Mashek and Coleman [18] found that the overexpression of ACS4, which is located on the mitochondrial-associated membrane in hepatocytes, increases EPA incorporation into cell lipids by 67% during a 3-h labeling period in COS-1 cells. MAG-lipase and α/-β-hydrolase domain 6 (ABHD6) and ABHD12 are enzymes which are also involved in intracellular degradation of MAG in many tissues. Poursharifi et al. [19] showed that the suppression of ABHD6 caused the accumulation of 1-MAG-containing saturated FA in pancreatic islets and INS-1 cells and also in white and brown adipose tissues, while the changes in 2-MAG content was modest. Gadja and Storch [20] suggest an important role on this subject for a liver-FA binding protein (LFABP), which is part of a complex along with microsomal TAG transport protein (MTP), CD36, and ApolipoproteinB48 (ApoB48), and is responsible for budding of prechylomicron transport vesicles (PCTV) from the endoplasmic reticulum (ER). Circulating FA and MAG enter the enterocyte via the basolateral membrane where they can be bound to LFABP, and bloodstream-derived FA and MAG are primarily oxidized or incorporated into phospholipids. Taken together, all this information supports the fact that for MAG there is a mediated transport across the cell membrane of enterocytes.

Currently, there is a growing research effort focused on the production of functional nutrients such as structured lipids. However, few studies have focused on assessing the effects of ARA- and DHA-MAG in colon cancer cells and the related molecular mechanisms. In a previous study focused on FFA, we demonstrated that DHA-FFA inhibited HT-29 cells proliferation to a higher extent than ARA-FFA did, with either proteasome or DNA replication, respectively, being the main mechanisms affected [21]. Here, we used the same cellular model, study design, and technology to investigate the actions of DHA and ARA-MAG in colorectal cancer (CRC) cells, and the results are compared with those reported for the corresponding FFA. For this objective, cell viability and cell cytotoxicity assays have been performed, and the biological pathways that are affected by these two MAGs have also been studied by means of sequential window acquisition of all theoretical mass spectra (SWATH)—mass spectrometry (MS) global protein quantitation followed by pathway analysis.

## 2. Materials and Methods 

### 2.1. Oil Samples and Purification of MAG

DHASCO^®^ (40% DHA, a mixture of the oil extracted from the unicellular alga *Crypthecodinium cohnii* and high oleic sunflower oil) and ARASCO^®^ (40% ARA, a mixture of an oil extracted from the unicellular fungi *Mortierella alpina* and high oleic sunflower oil) oils were supplied by Martek Bioscience Corporation (Columbia, MD, USA). Purification of ARA- and DHA-MAG was carried out according to the methodology described by González-Fernández et al. [22] based on a chromatography process. Briefly, both DHASCO^®^ and ARASCO^®^ oils were subjected to an enzymatic hydrolysis with porcine lipase. Then, MAGs were separated from the remaining hydrolysis products (mainly FFA and glycerol) using an open chromatography column with silica gel as stationary phase and a hexane/acetone mixture as mobile phase. Once MAG mixtures were obtained, another open chromatography column with silver nitrate as stationary phase was used to purify either DHA-MAG or ARA-MAG. Liquid chromatographic fractions were collected in test tubes and analyzed by gas chromatography (GC) to determine the purity grade according to Rodríguez-Ruiz et al. [23]. To this end, about 1 mg of each fraction was weighed into test tubes next to 1 mL of *n*-hexane and 1 mL of freshly prepared transesterification reagent (methanol and acetyl chloride 20:1 v/v). Then, the tubes were placed in a thermoblock at 100 °C for 30 min. After that, the mixtures were cooled at room temperature and 1 mL of distilled water was added. Samples were shaken and centrifuged (2500 rpm, 3 min) and the upper *n*-hexane layer collected and stored in numbered vials at −20 °C until GC analysis. The equipment used for FA methyl esters (FAME) analysis was a GC device (Focus, Thermo Electron, Cambridge, UK) equipped with flame ionization detector (FID) and an Omegawax 250 capillary column (30 m × 0.25 mm ID × 0.25 μm film thickness) (Supelco, Bellefonte, PA, USA). The oven temperature program was 90 °C (1 min), 10 °C/min to 100 °C (3 min), 6 °C /min to 260 °C (5 min). The injector temperature was 250 °C with split ratio 50:1, and injector volume was 4 μL. The detector temperature was 260 °C. The flow of carrier gas (N_2_) was 1 mL/min. Peaks were identified by retention times obtained for known FAME standards (PUFA No. 1, 47033; methyl linoleate 98.5% purity, L6503; and methyl stearidonate 97% purity, 43959 FLUKA) from Sigma, (St. Louis, USA), and FA contents were estimated by using methyl pentadecanoate (15:0; 99.5% purity; 76560 Fluka) from Sigma as internal standard.

### 2.2. Cell Assays

All assays were accomplished using the HT-29 colon cancer cell line, which was supplied by the Technical Instrumentation Service of University of Granada (Granada, Spain). Cell cultures and cell assays, that is, the 3-(4,5-dimethylthiazol-2-yl)-2,5-diphenyltetrazolium bromide (MTT) test, lactate dehydrogenase (LDH) assay and caspase-3 activity, were performed as previously described [21]. The number of cells used in the cell assays ranged from 5 × 10^3^ for LDH assay to 1 × 10^7^ for caspase-3 assay, as previously described [21,24].

### 2.3. SWATH-MS Differential Abundance Proteomics Analysis

HT-29 cells, cultured in media supplemented with 600 µM of either DHA- (*n* = 6) or ARA-MAG (*n* = 6) at 24 h, and the same cells with no acyl species added (control group, *n* = 6) were recovered and lysed. Protein extracts were obtained, and protein was precipitated with TCA/acetone for removing contaminants. Then, 40 µg protein for each sample were subjected to trypsin digestion, and massive protein relative quantitation was assessed following a SWATH approach as described in Ortea et al. [21]. Briefly, this approach consisted on three steps: (i) an MS/MS peptide library was built from the peptides and proteins identified in data-dependent acquisition (DDA) shotgun nanoLC-MS/MS runs from the samples, using Protein Pilot software (v5.0.1, Sciex) with a human Swiss-Prot protein database (20,200 protein entries, appended with the RePliCal iRT peptides (PolyQuant GmbH, Bad Abbach, Germany) and downloaded from UniProt on March 2017). Main settings used in Protein Pilot were iodoacetamide as Cys alkylation, trypsin as enzyme, TripleTOF 5600 as instrument, and thorough ID as search effort. The false discovery rate (FDR) was set to 0.01 for both peptides and proteins; (ii) each sample was analyzed with a variable SWATH LC-MS method; and (iii) protein quantitative data for the proteins contained in the peptide library were obtained from the SWATH runs by extracting the corresponding fragment ion chromatograms using the MS/MS^ALL^ with SWATH Acquisition MicroApp (v.2.0, Sciex). Peptide retention times were calibrated in all the SWATH runs using the RePliCal iRT peptides, spiked into each sample according to manufacturer’s instructions. To be confident on the proteins being identified and quantified, only those showing confidence scores above 99% and FDR below 1% were included in the analysis. For both kinds of LC-MS analysis, DDA and SWATH, a hybrid Q-TOF mass spectrometer (Triple TOF 5600+, Sciex, Redwood City, CA, USA) coupled on-line to nano-HPLC (Ekspert nLC415, Eksigent, Dublin, CA, USA) was used. For higher sensitivity, both DDA and SWATH runs were performed at nano-flow (300 nL/min) in a 25 cm long × 75 µm internal diameter column (Acclaim PepMap 100, Thermo Scientific, Waltham, MA, USA) using a 120 min gradient from 5% to 30% B (A: 0.1% FA in water; B: 0.1% in ACN).

### 2.4. Pathway and Gene Ontology (GO) Analysis

Advaita Bio’s iPathwayGuide (Advaita Corporation, Plymouth, MI, USA) was used for analyzing the significantly impacted pathways and for GO analysis. We considered a restrictive scenario, namely a differential expression threshold of log (fold change) 1.0 (that is, fold change 2.0) and adjusted *p*-value 0.01, in order to have more confidence in selecting the proteins that presented real expression changes. Data were analyzed in the context of pathways obtained from the Kyoto Encyclopedia of Genes and Genomes (KEGG) database (Release 78.0+/06-02, Jun 16).

### 2.5. Validation by Parallel Reaction Monitoring (PRM) Analysis

Several protein changes corresponding to the main significant affected pathways were subjected to validation using targeted quantitation by micro-HPLC PRM on a Triple TOF 5600+ (Sciex). Skyline software (v4.2.0) [25] was used for designing and optimizing a PRM acquisition method for all of the targeted proteins. Two to six proteotypic peptides for each protein were selected according to the following criteria: (i) enzyme: trypsin [KR/P] with 0 missed cleavages; (ii) 7–16 amino acid residues; (iii) carbomidomethylation of cysteines as structural modification; (iv) excluding peptides containing methionine; and (v) excluding the N-terminal amino acids. Transitions were filtered according to the following criteria: (i) +2 and +3 precursor charges; (ii) *y* and *b* product ion types; (iii) product ions from (m/z > precursor)—1 to last ion; (iv) method match tolerance 0.055 m/z; (v) a maximum of 10 product ions; and (vi) resolving power of 15,000 for MS/MS filtering. The HPLC gradient consisted of 5–22% buffer B (A: 0.1% FA in water; B: 0.1% FA in ACN) at 5 µL/min for 45 min, plus 5 min at 95% B and another 6 min at 5% B for re-equilibration. The column used was a 15 cm long × 300 µm internal diameter C18 column (Dionex Benelux B.V., Amsterdam, Netherland). Five to six individual samples (4 µg per sample) from each of the groups, DHA-MAG, ARA-MAG, and control, and two blank samples, were analyzed with the developed PRM, and the resulting chromatograms for all the monitored peptides were imported into Skyline and manually curated. Three injection replicates were used for calculation of the coefficients of variation (CV) for each of the monitored peptides.

### 2.6. Statistical Analysis

For cell assay results, statistical significance was determined using generalized linear models (GZLMs) using Statgraphics Plus 4.0 (Statistical Graphics Corp., Rockville, MD, USA). For SWATH-MS protein quantitation, data were analyzed following Ortea et al. [21]. Briefly, quantitative data were normalized for inter-run variability, and differences in protein abundance were assessed by applying a Student’s *t*-test, checking for multiple testing underestimation of *p*-values by obtaining a q-value estimation for FDR using the qvalue R package [26]. For impact pathway and GO analysis, iPathwayGuide software calculated a *p*-value using a hypergeometric distribution. The *p*-values were adjusted using FDR correction for pathways and Bonferroni correction for GO analysis. For PRM validation of protein changes, statistical analysis was performed using the ‘group comparison’ function in Skyline. Briefly, Skyline performed pairwise group comparisons for each protein using a Student’s *t*-test on the log2 transformed summed transition peak area for all the peptides from that protein, adjusting the *p*-values for multiple testing with the Benjamini-Hochberg correction. 

## 3. Results

### 3.1. DHA- and ARA-MAG Showed Differential and Concentration-Dependent Effects on HT-29 Cell Viability, Cell Membrane Integrity, and Apoptosis

The present study was conducted using the well-established HT-29 human colon cancer cell line. The purities of the assayed MAGs were 98.0% and 98.7% for DHA and ARA, respectively. According to González-Fernández et al. [22], during MAG purification process an acyl migration occurs quickly, so the concentrations of 1(3)-MAG balance with 2-MAG at one point that depends on several causes, e.g., solvent type, pH, and temperature. Although the purified forms are mainly 2-MAG and these are those added to cell cultures, in the culture medium the acyl-migration continues [27]. For this reason, we generically refer to MAG instead of 2-MAG.

**Figure 1:**
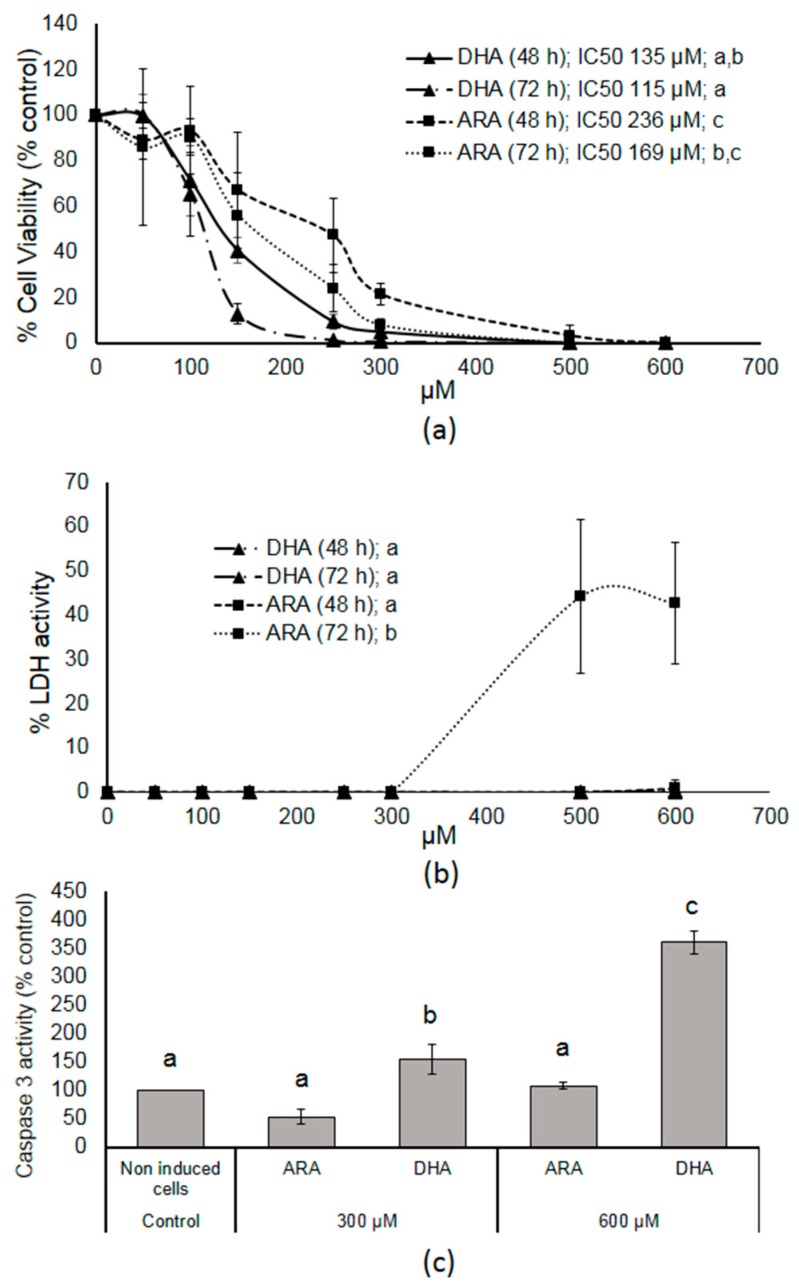
Plots showing results of cell assays. (**a**) Dose-dependent viability of HT-29 cells after exposure to docosahexaenoic acid (DHA)- and arachidonic acid (ARA)-monoacylglycerol (MAG). (**b**) Dose-dependent lactate dehydrogenase (LDH) release from HT-29 colon cancer cells after exposure to DHA- and ARA-MAG. (**c**) Dose-dependent caspase-3 activity from HT-29 colon cancer cells in comparison with untreated cells (control). Data represent the mean of three complete independent experiments ± SD (error bars). Data were analyzed using generalized linear models (GZLMs). There are no significant differences (*p* < 0.05) among series sharing the same letter.

First, we tested the actions of ARA- and DHA-MAG in HT-29 cells. Cell cultures were treated with different concentrations (50–600 μM) of ARA- and DHA-MAG for 48 and 72 h and then the MTT assay was performed to measure cell viability and cell proliferation (Figure 1a). After 48 and 72 h of treatment, the MTT assay revealed concentration-dependent inhibitory effects on HT-29 cells for both assayed acyl species. Cell growth inhibition was exercised much better by DHA-MAG, with IC_50_ values of 135 and 115 µM for 48 and 72 h, respectively, while for ARA-MAG these values were 236 and 169 µM. 

The actions of ARA- and DHA-MAG on cell membrane integrity measured by the LDH assay after 48 and 72 h of treatment, are shown in Figure 1b. The test assesses the release of the LDH enzyme into the culture medium after cell membrane damage caused by MAG. The tested concentrations ranged from 50 to 600 μM. No effect of DHA-MAG on the amount of LDH release was noted, while a 40% increase in LDH activity after 72 h treatment was detected at the highest ARA-MAG concentrations.

To clarify whether ARA- and DHA-MAG were able to reduce cancer cell viability by promoting apoptotic cell death, a classical marker of apoptosis, caspase-3, was determined. In this study, caspase activation was evaluated in cells treated with ARA- and DHA-MAG at 300 and 600 μM for 90 min (Figure 1c). Caspase-3 activity is expressed as the percentage of activity compared to that of the untreated samples. As shown, a significant increase (up to 361%) of caspase-3 activity in the HT-29 cells was observed after 90 min exposure to DHA-MAG, while ARA-MAG did not show remarkable effects as compared to the respective untreated controls.

### 3.2. SWATH Quantitation of 1882 proteins Showed That DHA- and ARA-MAG Differentially Affect the Whole Proteome of HT-29 Cells

Samples were analyzed by DDA nanoLC-MS/MS, and runs were searched against a human protein database using Protein Pilot software. As a result, after integrating all three data sets, 2140 proteins and 15,406 peptides were identified (FDR < 1% at both protein and peptide levels); the list of identified proteins is shown in Table S1. The identified MS/MS spectra were compiled into a spectral library containing 2002 proteins. Using this library, chromatographic traces were extracted from the SWATH runs for 7653 peptides, corresponding to 1882 proteins. SWATH-based quantification normalized data for these 1882 proteins in all the samples is shown in Table S2. 

**Figure 2:**
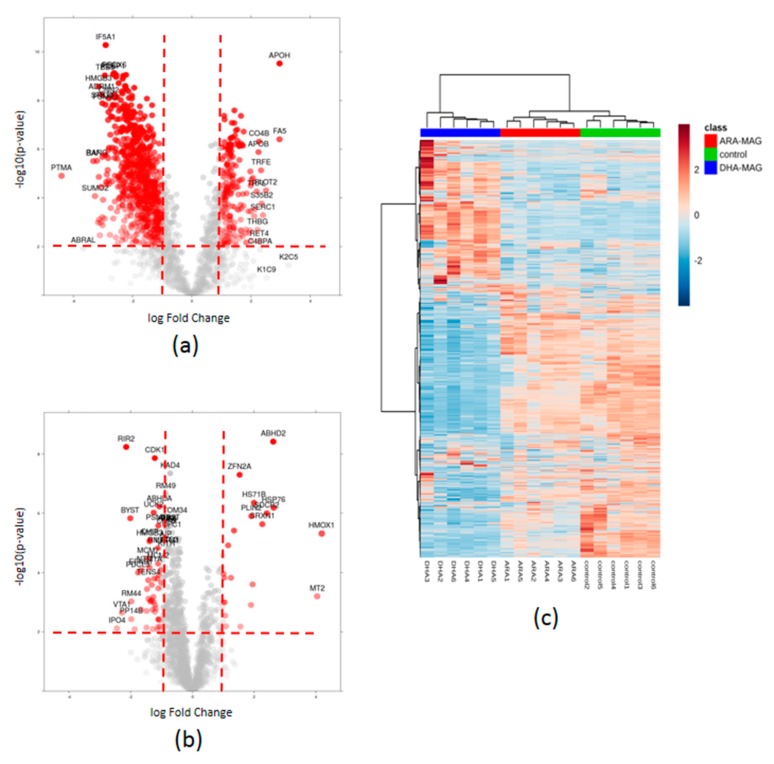
Quantitative proteomics result overview. Volcano plot showing proteins with differential expression as a result of the addition of (**a**) DHA-MAG; and (**b**) ARA-MAG. Red dotted lines show *p*-value < 0.01 and two-fold change cut-offs; proteins above these thresholds are shown in red. (**c**) Heat map including all 1882 quantified proteins; samples treated with DHA-MAG and ARA-MAG are separated from each other and from the control group.

Tables S3 and S4 show the results for the differential abundance tests for DHA-MAG vs. control and for ARA-MAG vs. control, respectively. For subsequent analyses, we considered a restrictive scenario, namely *p*-value < 0.01 and two-fold change (FC), in order to have more confidence in selecting the proteins presenting actual expression changes. When addressing the changes in HT-29 cell proteome caused by DHA-MAG, a total of 896 proteins showed changes in expression (189 up-regulated and 707 down-regulated) (Figure 2a). Applying the same *p*-value and FC thresholds, only 70 proteins revealed a differential abundance as a consequence of the exposure to the ARA-MAG supplemented medium, 21 proteins being up-regulated and 49 down-regulated (Figure 2b). When looking for the largest effects, extreme FCs (above 5.0) were found in 119 and six proteins (*p*-value < 0.01) for DHA- and ARA-MAG, respectively (Tables S3 and S4, respectively). Therefore, it is clear than DHA-MAG produces a deeper effect than ARA-MAG on HT-29 cancer cells. When comparing the differentially abundant proteins (*p*-value < 0.01 and FC **≥** 2.0) in both tested groups, only 45 proteins were commonly being affected. Multivariate analyses of SWATH-based data including all 1882 quantitated proteins showed a complete separation of all groups: DHA-MAG, ARA-MAG, and control (Figure 2c and Figure S1).

### 3.3. Pathway and GO Analysis Showed Different Mechanisms of Action of DHA- and ARA-MAG on HT-29 cells

**Figure 3:**
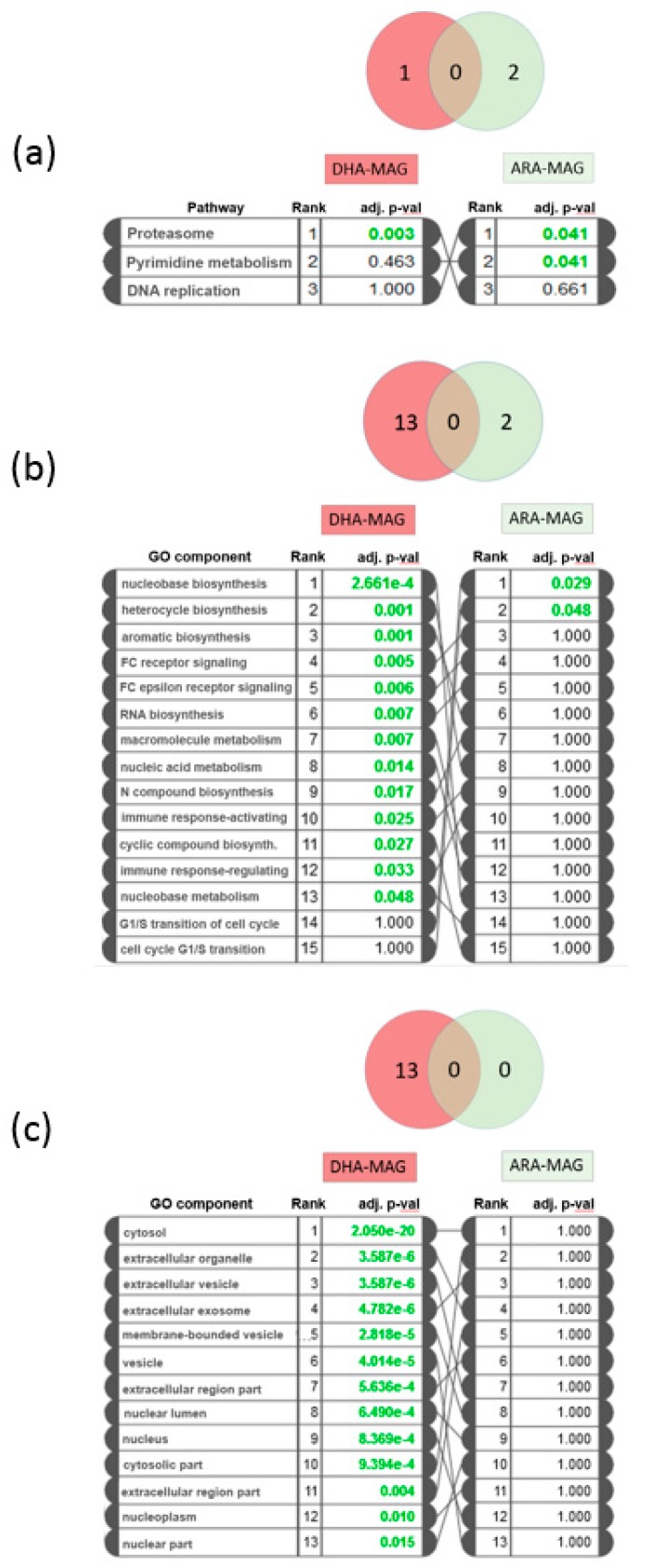
Pathway impact and gene ontology (GO) analysis for DHA-MAG and ARA-MAG. (**a**) Significantly impacted pathways and their associated adjusted *p*-values. (**b**,**c**) GO analysis showing significant (adjusted *p*-values < 0.05) (**b**) biological processes or (**c**) cellular components.

**Figure 4:**
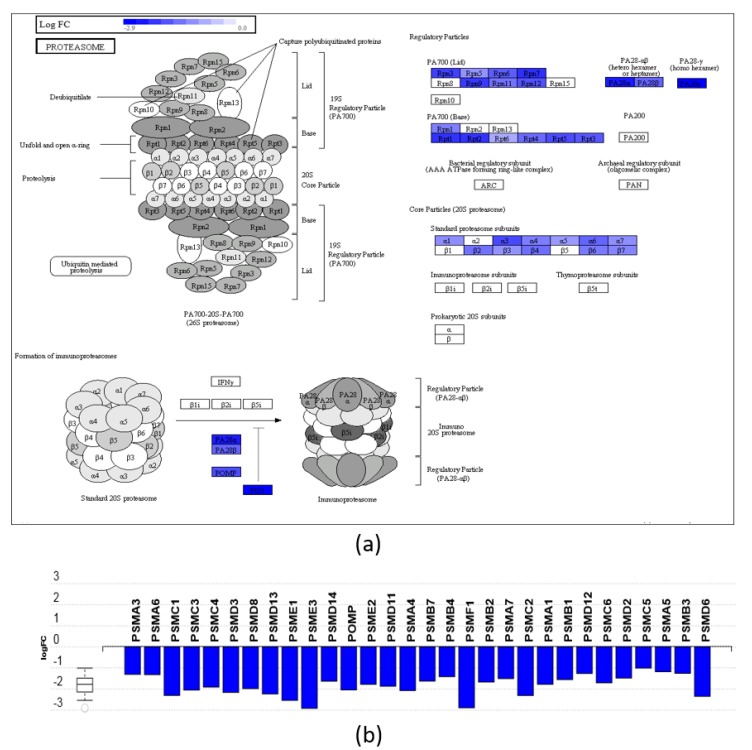
Effect of DHA-MAG extract on HT-29 cells’ proteasome pathway. (**a**) Proteasome pathway diagram highlighting regulated proteins. (**b**) Gene perturbation bar plot for affected proteins of the proteasome pathway.

**Figure 5:**
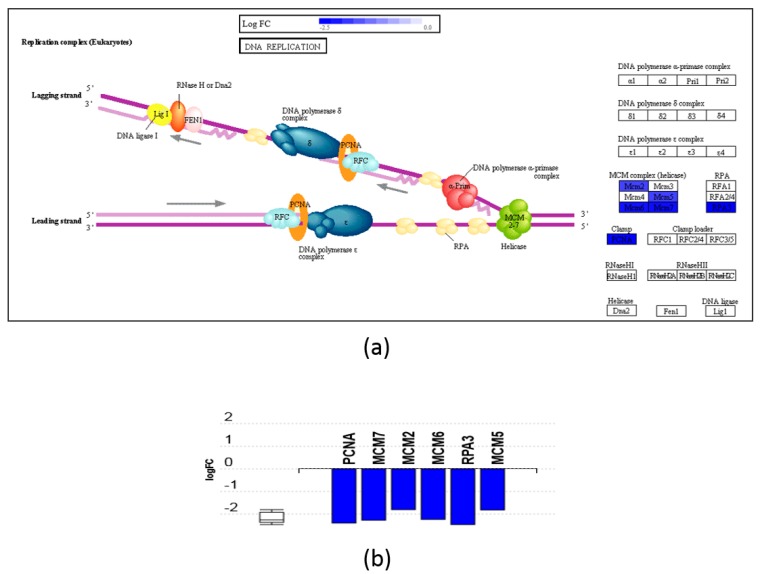
Effect of ARA-MAG extract on HT-29 cells’ DNA replication pathway. (**a**) DNA replication pathway diagram highlighting regulated proteins. (**b**) Gene perturbation bar plot for affected proteins of the DNA replication pathway.

The affected pathways and GO components were analyzed using iPathwayGuide software. Significantly impacted pathways and over-represented GO groups according to this analysis are shown in Figure 3. After FDR correction, only one pathway was found to be significantly impacted by DHA-MAG, namely the proteasome pathway (KEGG: 03050), with 30 proteins down-regulated (Figure 4). On the other hand, two pathways resulted as significantly affected by ARA-MAG, DNA replication (KEGG: 03030), with a DNA polymerase and three helicase subunits down-regulated (Figure 5); and pyrimidine metabolism (KEGG: 00240).

The biological processes GO component most affected by DHA-MAG was nucleobase-containing compound biosynthetic process (adjusted *p*-value 2.66 × 10^−4^) (Figure 3b), with 239 proteins presenting differential abundance (Table S5). For ARA-MAG, the only significantly over-represented biological processes according to our GO analysis were those related to the G1/S transition of the cell cycle (Figure 3b), presenting eight proteins being regulated (Table S5). Regarding GO analysis of cellular components, DHA-MAG regulated proteins were mainly related to cytosol (adjusted *p*-value 2.05 × 10^−20^), but also from extracellular and nuclear origin (Figure 3c and Table S6), while no cellular component resulted as over-represented as a consequence of ARA-MAG regulation.

### 3.4. SWATH Proteomics Analysis was validated by PRM

**Figure 6:**
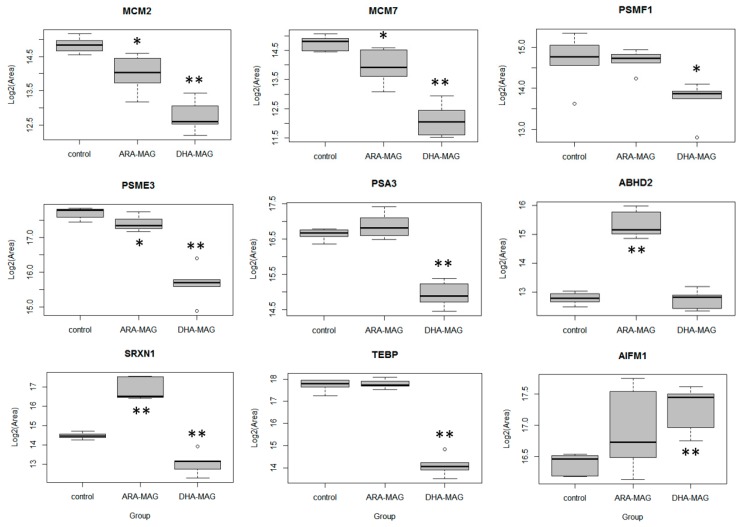
Differential expression of nine proteins from DHA-MAG- and ARA-MAG-treated HT-29 colorectal cancer cells by PRM quantification (* adjusted *p*-value <0.05, ** adjusted *p*-value <0.01; group comparison to control).

We developed a micro-HPLC PRM method to validate several of the protein changes previously found with the SWATH quantitative analysis workflow. Specifically, nine proteins were included in the PRM assay (Table 1): the most relevant pathways, as found in the pathway analysis, were represented by proteins MCM2 and MCM7 (helicase proteins from DNA replication pathway) and PSMF1, PSME3, and PSA3 (proteasome pathway); AIFM1 (apoptosis-inducing factor 1), as an apoptotic marker which we had found up-regulated in the SWATH analysis only in DHA-MAG treated cells but not in ARA-MAG, was also included in the validation assay; three other proteins showing SWATH-measured differences according to the compound used (ABHD2, up-regulated in ARA-MAG and not-significant in DHA-MAG; SRXN1, up-regulated in ARA-MAG and down-regulated in DHA-MAG; and TEBP, down-regulated in DHA-MAG and not-significant in ARA-MAG) were also included for validation of the SWATH results. After optimization of the method for these nine proteins, a total of 27 proteotypic peptides were used for targeted PRM validation (Table S7). As an indication of the precision of the PRM assay, measured CVs were below 20% for all the peptides except for peptide TFVDYAQK (CV of 22%); median CV for the entire peptide set was 9.0% (Figure S2 and Table S7). The effect of both compounds, DHA-MAG and ARA-MAG, on the nine targeted proteins, as measured by PRM, is shown in Figure 6.

## 4. Discussion

IC_50_ values obtained by the MTT assay for ARA- and DHA-MAG are lower than those obtained for both LCPUFAs in the FFA form applied to the same cell line [21]. These differences may be related to the existence of a protein-mediated transport for PUFA across cell membranes. In this regard, a specific protein-mediated process has been reported for intestinal Caco-2 cells, which allows the entry of LCPUFA into such cells [5], and both acyl forms, i.e., FFA and MAG, establish competition for the same membrane-associated protein transporters (FAT, FATP, CD36, FABP). The findings of this work outline other ones from Ramos Bueno et al. [24], who demonstrated that unspecific oil-derived ARASCO^®^- and DHASCO^®^-MAG induced noticeable in vitro antitumor activity on HT-29 cells. Previous studies demonstrated that DHA significantly decreases cancer cells proliferation [28]; while Pompeia et al. [12] found dose- and time-dependent ARA-induced cytotoxicity in leukocytes, i.e., ARA at 10–400 µM induced apoptosis, while at concentrations above 400 µM the noted effect was necrosis. Several studies have discussed the relationship between FAs and mitochondrial permeability transition. Such relation is modulated by a variety of effectors of cell death, including reactive oxygen species (ROS), which are important messengers in normal-cell signal transduction and cell cycle being produced by mitochondria after stimulation of the TNFα receptor [29,30]. Moreover, Scorrano et al. [29] showed that ARA is a powerful permeability-transition inducer in MH1C1 cells, causing a release of cytochrome c followed by cell death.

The LDH test is a colorimetric method suitable for the measurement of cell membrane integrity. It is based on the measurement of LDH enzyme activity, whose increase in the culture supernatant is proportional to the number of lysed cells [31]. Until now, several authors have studied cell membrane permeability to determine non-cytotoxic concentrations of MAG on different cell lines, such as Caco-2 and HT-29 cells, and no significant toxicity measured by the LDH assay was observed [15,24,32,33]. In this study, toxicity was found only after 72-h treatment at the highest ARA-MAG concentrations. This might be linked to the differential spatial configuration of both ARA- and DHA-MAG. The activities of both ARA and DHA were previously checked in the FFA form, with a higher activity being noted for ARA than for DHA [21,34], thus, after the intracellular hydrolysis of both MAGs, the action of the ARA-MAG would still prevail. The low activity could be due to the fact that both ARA- and DHA-MAG cannot be integrated effectively into cell membranes, since MAGs are low-polarity compounds and therefore they cannot establish effective chemical links with membrane proteins on these structures [35]. In this regard, Dommels et al. [36] stated that the cytotoxic effects mediated by some PUFAs, e.g., ARA and EPA, are due to the peroxidation products generated during lipid peroxidation and cyclooxygenase activity. However, MAGs are known to be surface-active compounds, so they might show minor cytotoxic effects on cells by disrupting cell membranes [32]. 

Caspase-3 is considered to be the most important effector of apoptosis and a marker for both intrinsic and extrinsic pathways [37]. An important aspect to consider is the chemical structure of the different MAGs, which affects the potency to induce apoptosis; in this regard, Philippoussis et al. [3] concluded that SFA-based MAG had no effect on such phenomenon, while PUFA-MAGs were highly potent to induce apoptosis in T-cells. The apoptotic activity noted here for DHA agrees with previous findings [3,38,39], and the potency of both LCPUFA-MAGs was higher than that previously reported for both FFA-based LCPUFAs [21]. Our findings demonstrated that DHA-MAG induces apoptotic cell death via activation of caspase-3. The non-activation of caspase by ARA-MAG may be due to the chemical structure of MAG, since in the FFA form such activity was detected [21]. These results completely agree with those from Ho and Storch [5], who suggested the existence of a protein-mediated process for MAG transport through cell membranes. Accordingly, LCPUFA-based MAGs could reach high concentrations inside cells and would be able to perform effective apoptosis actions, as reported here.

SWATH, one of the recently developed data-independent acquisition (DIA) MS strategies [40], shows outstanding precision and accuracy even when used for proteome-wide quantitation [41]. SWATH performance is comparable to that of selected reaction monitoring (SRM), the golden standard for protein and small molecule quantitation [41]. SWATH consists of acquiring MS/MS data in stepped m/z fragmentation windows, and then matching the resulting fragment ions to peptides and proteins using a previously generated MS/MS spectral library, so fragment ion chromatograms can be in-silico extracted and used for label-free protein quantitation. Here we have used a SWATH v2.0 method, with variable Q1 isolation windows according to the ion density found in previous DDA runs, which has been described to improve peptide identification and quantification [42]. The results found in our SWATH analysis, with DHA-MAG producing a deeper effect than ARA-MAG on the HT-29 cancer cell proteome, suggest that the decrease of cell viability and increase of apoptosis observed should be produced by means of different mechanisms depending on the MAG tested. For comparing these results to those found in our previous work [21], it has to be noted that both experiments (cell cultures and cell assays) were run in parallel, and the analytical LC-MS methods and bioinformatics analysis performed have been exactly the same. All LC-MS runs were combined in one dataset, normalized for inter-run variability, and analyzed all together (SWATH extraction, pathways, and GO analysis). When comparing the results with those previously obtained for DHA- and ARA-FFA [21], 284 (45 up-regulated and 239 down-regulated) and 73 (27 up-regulated and 46 down-regulated) proteins, respectively, were reported for the FFA forms. Therefore, these previous results also showed a deeper effect for DHA than for ARA. Within the DHA-derived molecules, MAG affected the HT-29 cell proteome more globally than FFA did (896 vs. 284 differentially expressed proteins, respectively), although there is a protein core of 237 proteins that are common to both DHA forms (Figure S3). Taking all these figures together, these results indicates that (i) DHA and ARA (in both forms, FFA and MAG) differentially affect the whole proteome of HT-29 cells, suggesting that the decrease of cell viability and increase of apoptosis observed should be produced by means of different mechanisms depending on the molecule tested; and (ii) MAG have a much deeper effect than FFA only for DHA forms, not for ARA. As an additional interesting result, we found PTGES3 (prostaglandin E synthase 3, TEBP, accession Q15185) was strongly down-regulated in DHA-MAG (fold-change 0.14, that is, seven times less abundant in DHA-MAG than in the control group) (Table S3). This protein, also down-regulated as an effect of DHA-FFA (fold-change 0.5, that is, two-fold less abundant in DHA-FFA than in control) [21], belongs to the prostaglandin biosynthesis pathway, and catalyzes the conversion of prostaglandin H2 to prostaglandin E, that is, the next step to the transformation of arachidonic acid to prostaglandin H2, which is catalyzed by COX-2. COX-2 is involved in regulation of apoptosis and proliferation of colorectal, liver, pancreatic, breast, and lung cancer cells [43], and although we do not have quantitative data for COX-2 in our results, the strong down-regulation of PTGES3 we found could mean that one of the antitumor activities of DHA could be effected by means of the prostaglandin cascade, since it plays an important role in antigen presentation and immune activation in cancer [44]. 

The significantly affected pathways were analyzed using iPathwayGuide software, which implements an ‘impact analysis’ approach, taking into consideration not only the over-representation of differentially expressed (DE) genes in a given pathway (i.e., enrichment analysis), but also topological information such as the direction and type of all signals in a pathway, and the position, role, and type of each protein [45]. Only the proteasome pathway resulted as significantly impacted by DHA-MAG in our analysis. The proteasome is a large protein complex which main action is degrading ubiquitinated-labeled proteins [46], and plays an important role in the regulation of many cellular processes such as cell cycle, cell differentiation, signal transduction, inflammatory response, and antigen processing. In Figure 4, a diagram of the different particles and proteins constituting the proteasome are shown, highlighting the proteins that we have found to be significantly regulated as an effect of DHA-MAG on HT-29 cancer cells. In our results, we found 30 proteins from the proteasome pathways to be significantly down-regulated, comprising all the main particles: 11 proteins from the 20S core particle and 14 proteins from the 19S regulatory particle, in both lid and base subunits. In addition, the PA28-αβ and PA28-γ are also down-regulated. PA28-γ has been found in the nucleus and plays an important role in the regulation of apoptosis and cell cycle progression [47]. Interestingly, proteasome was the main pathway that was found to be affected by DHA-FFA in our previous study using the same workflow [21]. In that case, 18 proteins from the proteasome complex resulted as down-regulated. Since the number of DE proteins reached 30 in the case of DHA-MAG, we can conclude that both forms of DHA affect the proteasome, but the MAG form induces its massive switch-off. Interestingly, proteasome inhibitors, such as natural polyphenol compounds, have been tested in clinical trials as drug candidates for treating different cancers, due to their ability to induce apoptosis and reduce cell proliferation [48,49]. According to the strong down-regulation of the proteasome particles we have found in our study, we suggest DHA-derived MAG, in addition to DHA-FFA as previously reported, as one of these candidates that deserve further studies as an anticancer effector.

Two pathways resulted as significantly affected by ARA-MAG: DNA replication and pyrimidine metabolism. Regarding the DNA replication pathway, POLE3, one of the proteins conforming the DNA polymerase Ɛ, and three proteins from the helicase (MCM2, MCM3, and MCM7) were found to be significantly down-regulated (Figure 5). In addition, the remaining helicase proteins, MCM4, MCM5, and MCM6, which are not significant according to our threshold (above two-fold change), are also affected to a certain extent, since corresponding fold-changes (case to control) found are 0.51, 0.55, and 0.51, respectively. The DNA helicase protein complex is responsible for unwinding the duplex DNA helix ahead of the DNA synthetic machinery at the replication fork [50]. Since DNA replication is linked to cell cycle progression and to DNA repair processes, it would be expected that the down-regulation of the helicase-constituting proteins and POLE3 would have an inhibitory effect on these other related processes. Actually, our results show that cell cycle pathway (KEGG: 04110), even though is not significant in our pathway analysis (adjusted *p*-value of 0.214), is perturbed by the addition of the ARA-MAG extract, since several proteins belonging to this pathway are regulated by it (Figure S4a). The other pathway that resulted as significantly impacted according to our pathway analysis was the pyrimidine metabolism pathway (KEGG: 00240) (adjusted *p*-value 0.041), where ARA-MAG produces the under-expression of several proteins (Figure S4b). When comparing to the results obtained in the previous study using ARA-FFA, these two pathways, DNA replication and pyrimidine metabolism, together with cell cycle, also resulted as significantly impacted in the pathway analysis [21]. 

Interestingly, DHA-MAG also affects proteins from the DNA replication pathway (Figure S5), showing four helicase proteins being down-regulated, although our pathway analysis does not report this pathway as statistically significant due to the high global number of affected proteins. DNA replication turned out to be significantly impacted in our previous study using DHA-FFA, and therefore we can say that both forms of DHA induce a down-regulation of helicase proteins in addition to the deep effect on the proteasome. In contrast, ARA-MAG, as was the case also for ARA-FFA [21], does not induce a strong effect on the proteasome pathway (*p*-value of 0.661) since only two of the proteins included in this pathway were found to be regulated, PSMF1 and PSME3, with fold-changes (case to control) of 0.44 and 0.47, respectively (Figure S6).

For validating the SWATH-derived results, we developed a micro-HPLC PRM method which included 27 proteotypic peptides from a total of nine proteins. Interestingly, these nine proteins covered all the possible differences (up-regulated, down-regulated, or non-significant changes) for both comparisons (DHA-MAG to control and ARA-MAG to control) as found in the SWATH analysis. PRM [51], being a high-resolution (HR) MS targeted proteomics approach, has significantly greater sensitivity, accuracy, and precision than non-targeted discovery measurements, and more specificity than non-HR targeted methods, and therefore it is commonly used as a tool for validating protein abundance changes in all kinds of quantitative proteomics applications [52]. The results from the PRM assay were consistent with those previously found with the SWATH discovery approach (Table 1) for both comparisons (DHA-MAG to control and ARA-MAG to control), in terms of significance, fold-change, and direction of change (up- or down-regulation). Actually, the fold-changes were very similar in both measures, SWATH and PRM. As some examples, MCM2 fold-changes for the DHA-MAG to control comparison are 0.29 and 0.24, 0.40 and 0.38 for SRXn1, or 2.34 and 1.82 for AIFM1, for SWATH and PRM, respectively. For the comparison of ARA-MAG to control, some example fold-changes are 0.46 and 0.56 for MCM2, 4.81 and 5.12 for SRXN1, or 1.05 and 1.18 for PSA3 (Table 1). Only protein PSMF1 showed a different behavior when comparing SWATH and PRM analyses, and only for the ARA-MAG to control comparison (Table 1). While it had been found to be down-regulated by SWATH, PRM analysis found a fold-change of 1.0 (and, accordingly, no statistically significant difference). When inspecting the PRM results for this protein, we found that this issue could be explained by a discordance between the measures for the two peptides that were monitored for this protein: one of the peptides (ALIDPSSGLPNR) showed a fold-change of 0.56, while the fold-change for the other peptide (LPPGAVPPGAR) was 1.41 (Table S7). On the other hand, this discordance between the two peptides was not found in the comparison DHA-MAG to control, where the protein was found to be significantly down-regulated in both PRM and SWATH measures.

An interesting finding of the PRM analysis was the validation of the strong down-regulation of protein TEBP, prostaglandin E synthase 3, by DHA-MAG: measured fold-changes were 0.14 and 0.08 (SWATH and PRM, respectively), with adjusted *p*-values close to zero (Table 1), while ARA-MAG did not affect this protein. Prostaglandin E synthase 3 is one of the main proteins in the prostaglandin biosynthesis, converting prostaglandin H2 in prostaglandin E. Prostaglandin H2, the rate-limiting step in the formation of prostaglandins, is the product of prostaglandin G/H synthase 2, or COX-2, which has been related to colorectal cancer and whose inhibition (e.g., by nonsteroidal anti-inflammatory drugs) has been linked to tumor cell apoptosis, inhibition of proliferation, and reduction of colorectal cancer risk [53,54]. Therefore, it could be proposed that, apart from the proteasome pathway, one of the mechanisms contributing to the anticancer activity of DHA-MAG in HT-29 cells is carried out through the inhibition of prostaglandin synthesis, counteracting COX-2.

It should be noted here that an increase in lipid droplet accumulation, as a consequence of a potential saturation of the cells by lipids, could have a role in some of the effects found. However, we have demonstrated, and this is the main finding of this work, that, depending on the MAG, the molecular mechanisms working in the background are different, with DHA-MAG deeply affecting the proteasome, with 30 proteins being strongly regulated, while ARA-MAG, with only two proteins affected, does not. Actually, the conclusion of our study is that DHA- and ARA-MAG, while differentially affecting the whole proteome of HT-29 cancer cells by means of different mechanisms are not affected by the possibility of lipid droplets playing a role on the effect seen.

In summary, we have demonstrated that both ARA- and DHA-MAG showed concentration-dependent inhibitory effects on HT-29 cell viability, with a clear ability of DHA-MAG to induce cell death. The biological interpretation of SWATH-MS-generated proteomics data, validated by the quantification of nine relevant proteins by PRM, revealed that DHA-MAG outperforms the effect previously described for DHA-FFA, having a massive effect on the proteome of HT-29 cancer cells, with the proteasome complex being completely shut down. The strong down-regulation of prostaglandin E synthase 3, validated by PRM, also suggest a significant role of the prostaglandin synthesis in the anticancer activity of DHA-MAG in these colorectal cancer cells. On the other hand, although the effect of ARA-MAG is reduced in comparison to that of DHA-MAG, mainly in terms of inducing cell death, it still produces concentration-dependent inhibitory effects on HT-29 cell viability, as revealed by the MTT test. According to the proteomics experiments, this decrease of cell viability could be effected through inhibition of DNA replication and G1/S cell cycle transition, as it was previously described for ARA-FFA. Even though the MAG concentration used in the proteomics experiments (600 µM) could be considered relatively high, previous studies have reported higher (above 1700 µM) FA concentrations reached in human volunteers [55] and therefore, in the case of developing drugs based on MAG (and the corresponding FFA) for the treatment of cancer, 600 µM would be physiologically achievable, and it even would not represent negative effects on health, although, of course, further research in preclinical models and also in the clinical setting should be needed. 

## Figures and Tables

**Table 1:** Differential abundance for the proteins subjected to validation. Fold-changes and statistical significance are shown for both SWATH and Parallel Reaction Monitoring (PRM) analyses.

**Protein ID**	**Protein Name**	**ARA-MAG to Control**					
		**SWATH**			**PRM Validation**		
		**FC**	***q*-Value**	**Significant Change**	**FC**	**adj. *p*-Value**	**Significant Change**
MCM2	DNA replication licensing factor MCM2	0.46	8.3 × 10^−0^	↓	0.56	2.1 × 10^−2^	↓
MCM7	DNA replication licensing factor MCM7	0.37	6.4 × 10^−4^	↓	0.57	2.9 × 10^−2^	↓
PSMF1	Proteasome inhibitor PI31 subunit	0.44	7.4 × 10^−3^	↓	1.00	1.0 × 10^+0^	no
PSME3	Proteasome activator complex subunit 3	0.47	1.3 × 10^−4^	↓	0.81	3.7 × 10^−2^	↓
PSA3	Proteasome subunit alpha type-3	1.05	3.6 × 10^−1^	no	1.18	2.2 × 10^−1^	no
ABHD2	Abhydrolase domain-containing protein 2	6.14	2.4 × 10^−6^	↑	5.81	0	↑
SRXN1	Sulfiredoxin-1	4.81	1.3 × 10^−4^	↑	5.12	0	↑
TEBP	Prostaglandin E synthase 3	0.97	3.6 × 10^−1^	no	1.03	8.7 × 10^−1^	no
AIFM1	Apoptosis-inducing factor 1, mitochondrial	1.24	6.7 × 10^−2^	no	1.42	1.3 × 10^−1^	no
**Protein ID**	**Protein Name**	**DHA-MAG to Control**					
		**SWATH**			**PRM Validation**		
		**FC**	***q*-Value**	**Significant Change**	**FC**	**adj. *p*-Value**	**Significant Change**
MCM2	DNA replication licensing factor MCM2	0.29	4.8 × 10^−6^	↓	0.24	0	↓
MCM7	DNA replication licensing factor MCM7	0.21	2.6 × 10^−6^	↓	0.16	0	↓
PSMF1	Proteasome inhibitor PI31 subunit	0.13	1.8 × 10^−5^	↓	0.50	2.1 × 10^−2^	↓
PSME3	Proteasome activator complex subunit 3	0.13	1.4 × 10^−7^	↓	0.24	0	↓
PSA3	Proteasome subunit alpha type-3	0.40	6.7 × 10^−7^	↓	0.31	0	↓
ABHD2	Abhydrolase domain-containing protein 2	1.35	1.0 × 10^−1^	no	0.97	8.4 × 10^−1^	no
SRXN1	Sulfiredoxin-1	0.40	9.7 × 10^−5^	↓	0.38	7.0 × 10^−4^	↓
TEBP	Prostaglandin E synthase 3	0.14	2.0 × 10^−7^	↓	0.08	0	↓
AIFM1	Apoptosis-inducing factor 1, mitochondrial	2.34	1.6 × 10^−6^	↑	1.82	1.1 × 10^−3^	↑

## Supplementary Materials

The following are available online at https://www.mdpi.com/2072-6643/11/12/2984/s1, Table S1: List of identified proteins in the integrated data set. FDR was set to 1% at both peptide and protein levels. Table S2: SWATH-based protein quantification data for all the samples. 1882 proteins were quantified by SWATH-MS and measures were normalized for inter-run variability as described in Material and Methods section. Table S3: Differential abundance test for DHA-MAG vs. control. Fold changes, resulting *p*-values, and FDR analysis q-values for each of the 1882 quantified proteins are shown. Table S4: Differential abundance test for ARA-MAG vs. control. Fold changes, resulting *p*-values, and FDR analysis q-values for each of the 1882 quantified proteins are shown. Table S5: GO analysis: biological processes. Differential expressed proteins found in each biological process, together with all the proteins in that process, and the adjusted *p*-value, are shown for both comparisons, DHA-MAG vs. control and ARA-MAG vs. control. Table S6: GO analysis: cellular components. Differential expressed proteins found in each cellular component group, together with all the proteins in that component, and the adjusted *p*-value, are shown for both comparisons, DHA-MAG vs. control and ARA-MAG vs control. Table S7: Peptides selected for PRM validation. Fold-changes and adjusted *p*-values are shown for the comparisons DHA-MAG to control and ARA-MAG to control, together with the coefficient of variation (CV) measured using three technical replicates for each of the peptides. Figure S1: Multivariate analysis including all 1882 quantified proteins, showing the complete separation of the samples from each group (DHA-MAG, ARA-MAG, and control). (a) Group-averaged heat map; (b) partial least-squares discriminant analysis (PLS-DA); and (c) hierarchical cluster analysis (Spearman distance). Figure S2: Coefficient of variation (CV) for the 27 peptides monitored in the PRM validation, calculated using three injection replicates. Median CV for the PRM assay was 9%, with all peptides but one showing a CV below 20%. Figure S3: Differentially expressed proteins found as effect of DHA- and ARA-MAG, together with the previous results for DHA- and ARA-FFA [21]. Figure S4: Effect of ARA-MAG extract on HT-29 cells (a) cell cycle pathway (KEGG: 04110), highlighting protein perturbation according to our quantification results and showing coherent cascades; and (b) pyrimidine metabolism pathway (KEGG: 00240), highlighting significantly regulated proteins. Figure S5: Effect of DHA-MAG extract on HT-29 cells DNA replication pathway (KEGG: 03030). (a) DNA replication pathway diagram highlighting significantly regulated proteins. (b) Gene perturbation bar plot for DNA replication pathway affected proteins. Differentially expressed genes are represented with negative values in blue and positive values in red. In this case all proteins highlighted are under-regulated. Figure S6: Effect of ARA-MAG extract on HT-29 cells proteasome pathway (KEGG: 03050). (a) Proteasome pathway diagram highlighting significantly regulated proteins. (b) Gene perturbation bar plot for Proteasome pathway affected proteins. Differentially expressed proteins are represented with negative values in blue and positive values in red. In this case all proteins highlighted are under-regulated. The mass spectrometry proteomics data have been deposited to the ProteomeXchange Consortium via the PRIDE partner repository with the dataset identifier PXD014874.
