# Supplementary figures and images for: SWATH Differential Abundance Proteomics and Cellular Assays Show In Vitro Anticancer Activity of Arachidonic Acid- and Docosahexaenoic Acid-Based Monoacylglycerols in HT-29 Colorectal Cancer Cells
Source: Nutrients. 2019 Dec 6;11(12):2984. doi: 10.3390/nu11122984 (PMC6950369; doi:10.3390/nu11122984)

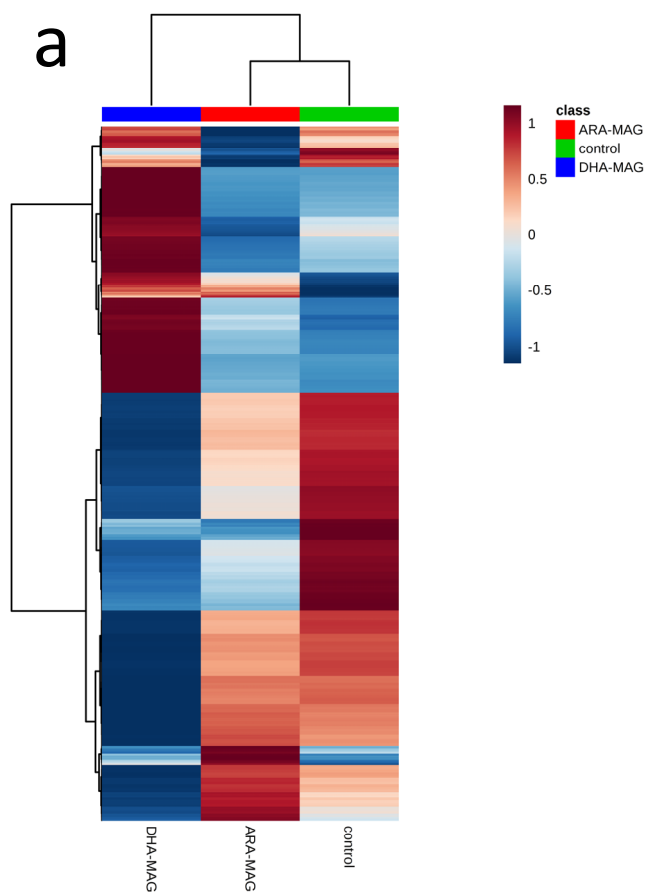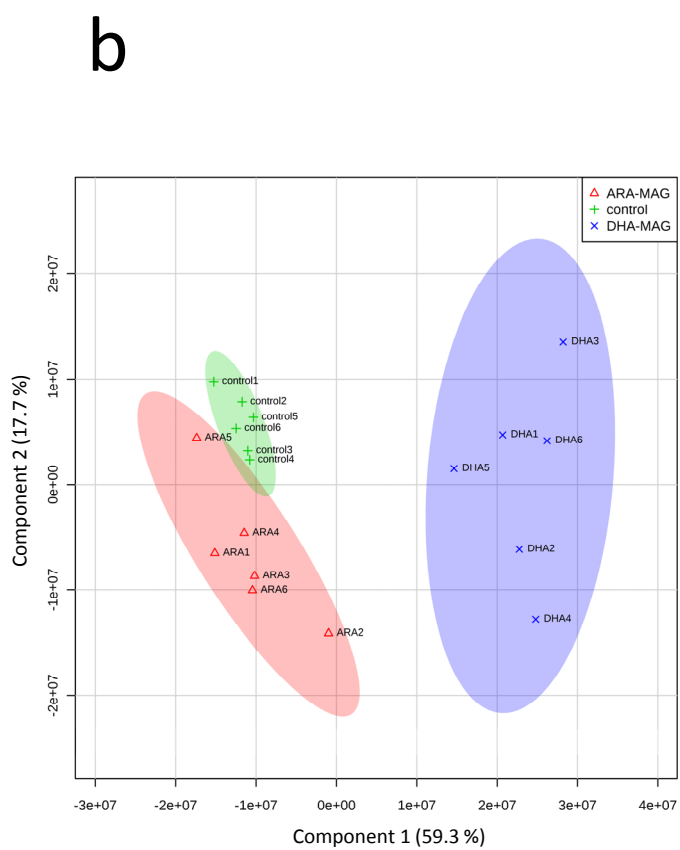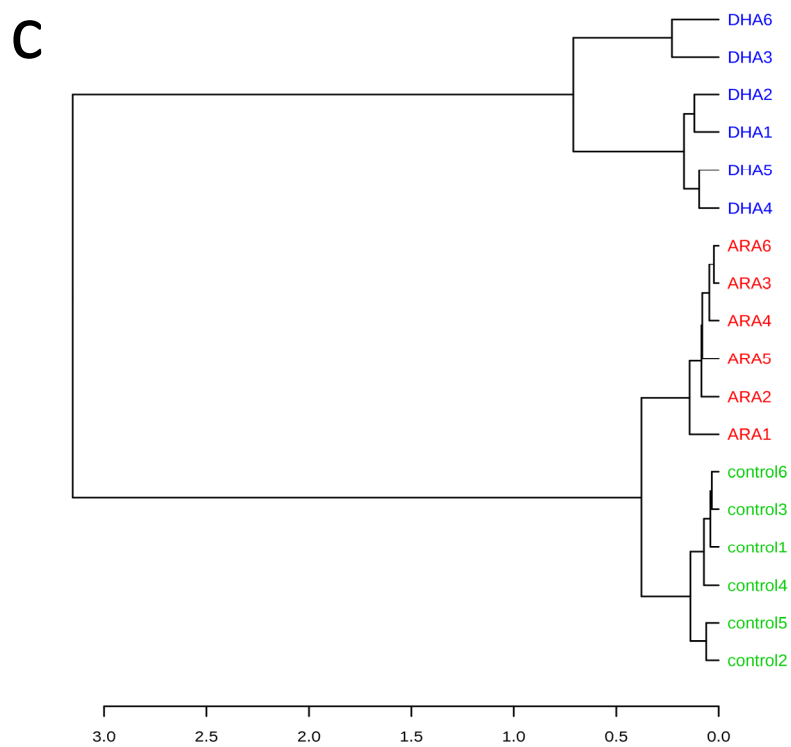

Supplement: Supplementary file 1 [file nutrients-11-02984-s001.zip › Figure S1.pdf]

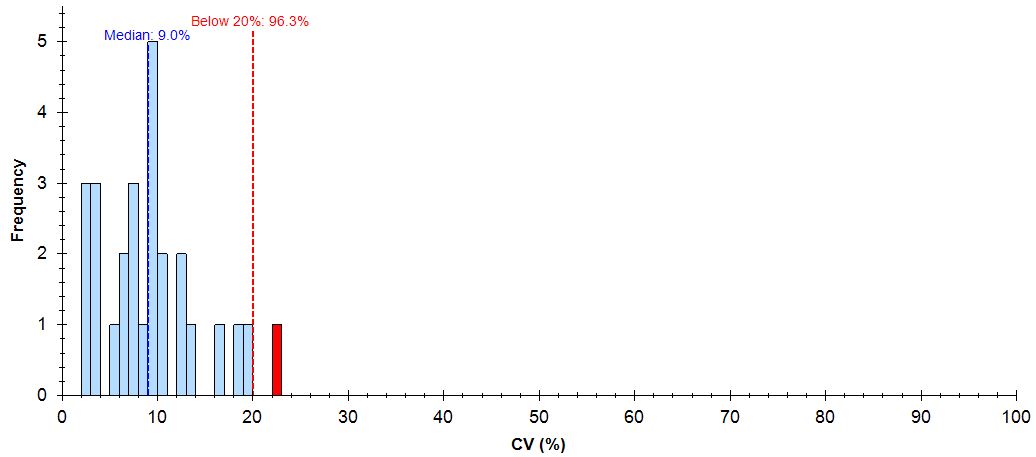

Supplement: Supplementary file 1 [file nutrients-11-02984-s001.zip › Figure S2.JPG]

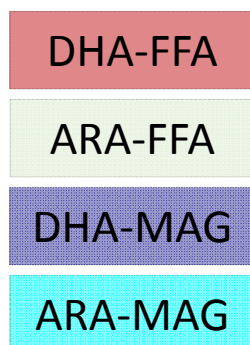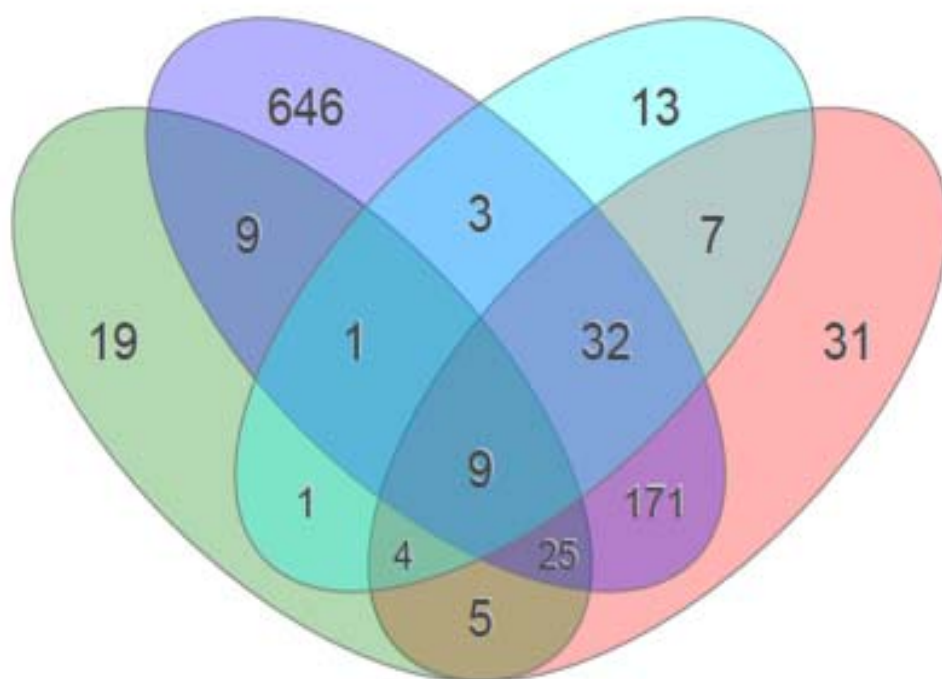

Supplement: Supplementary file 1 [file nutrients-11-02984-s001.zip › Figure S3.pdf]

a

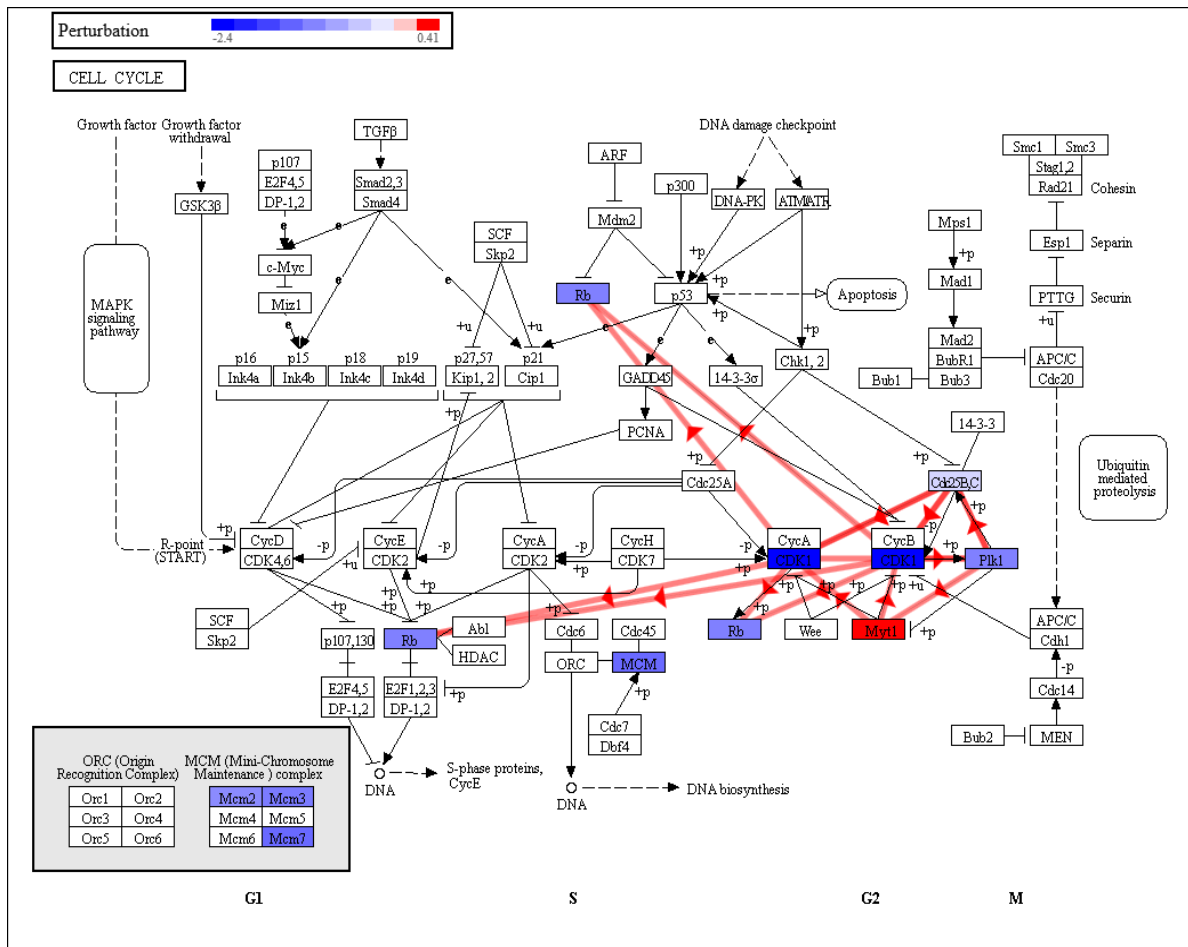

b

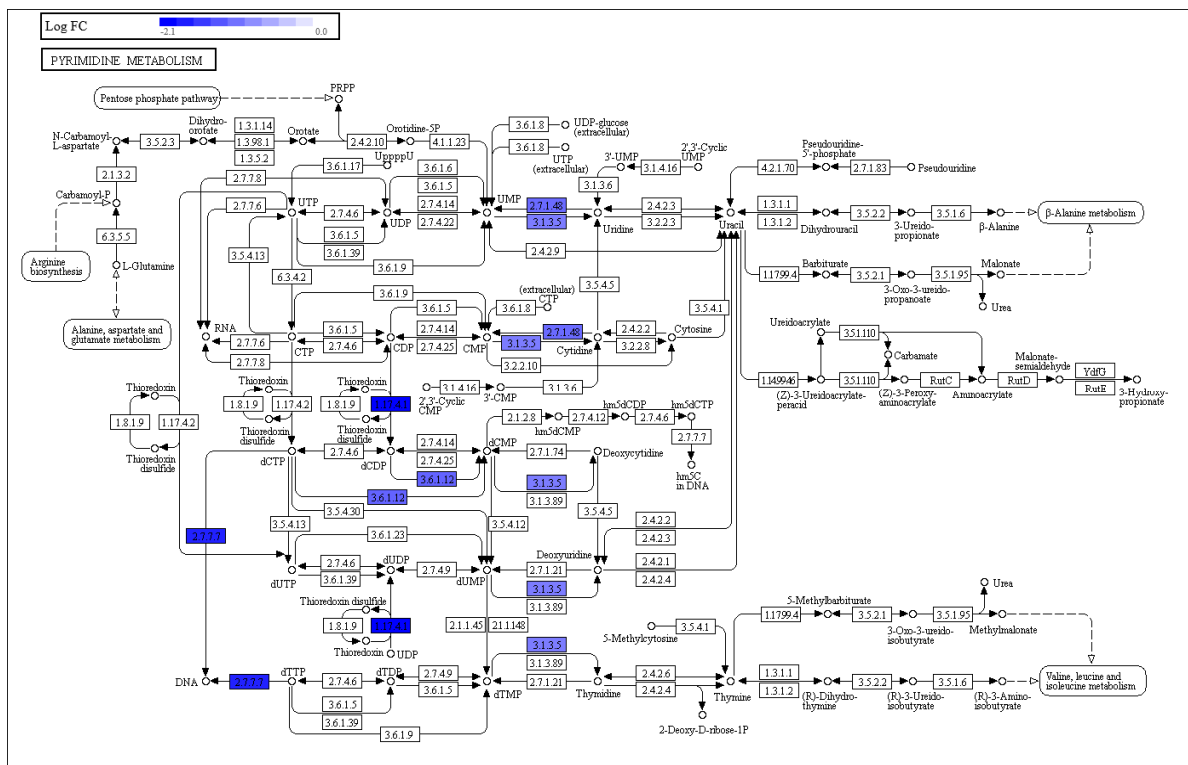

Supplement: Supplementary file 1 [file nutrients-11-02984-s001.zip › Figure S4.pdf]

a

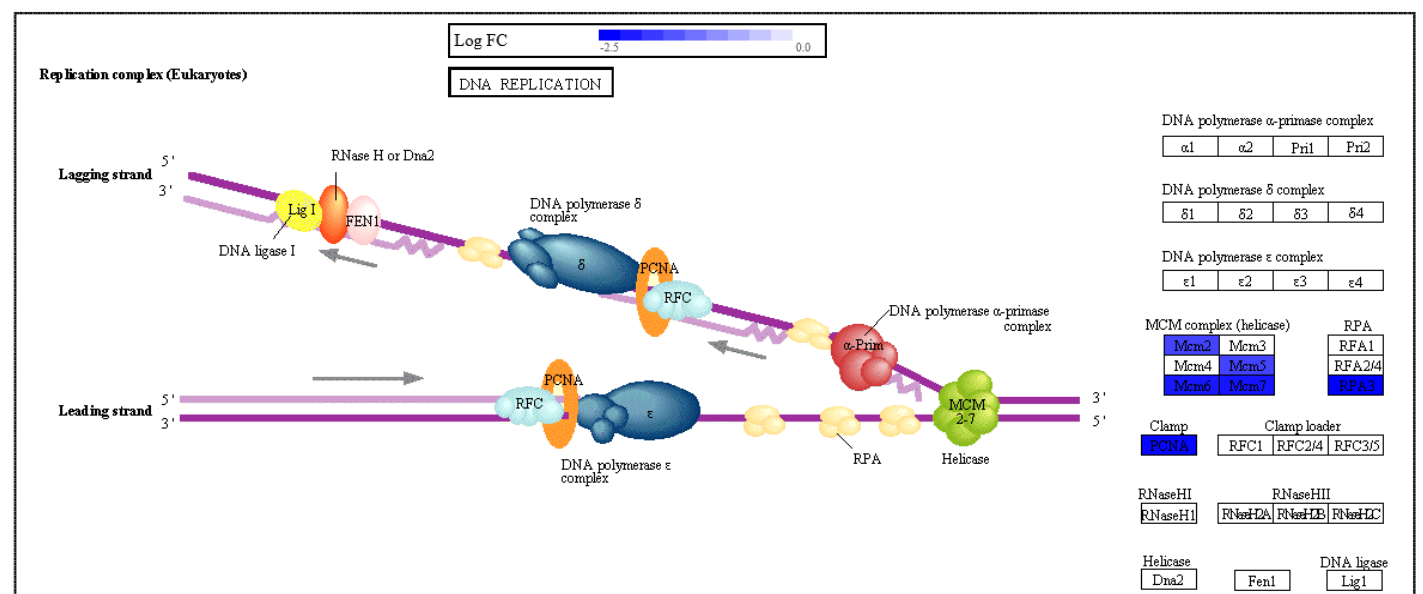

b

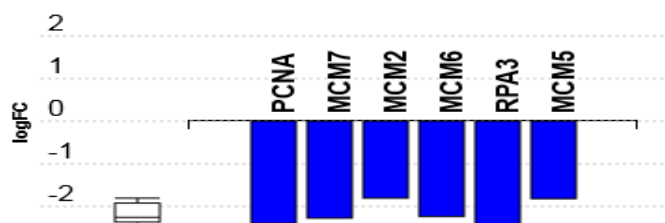

Supplement: Supplementary file 1 [file nutrients-11-02984-s001.zip › Figure S5.pdf]

a

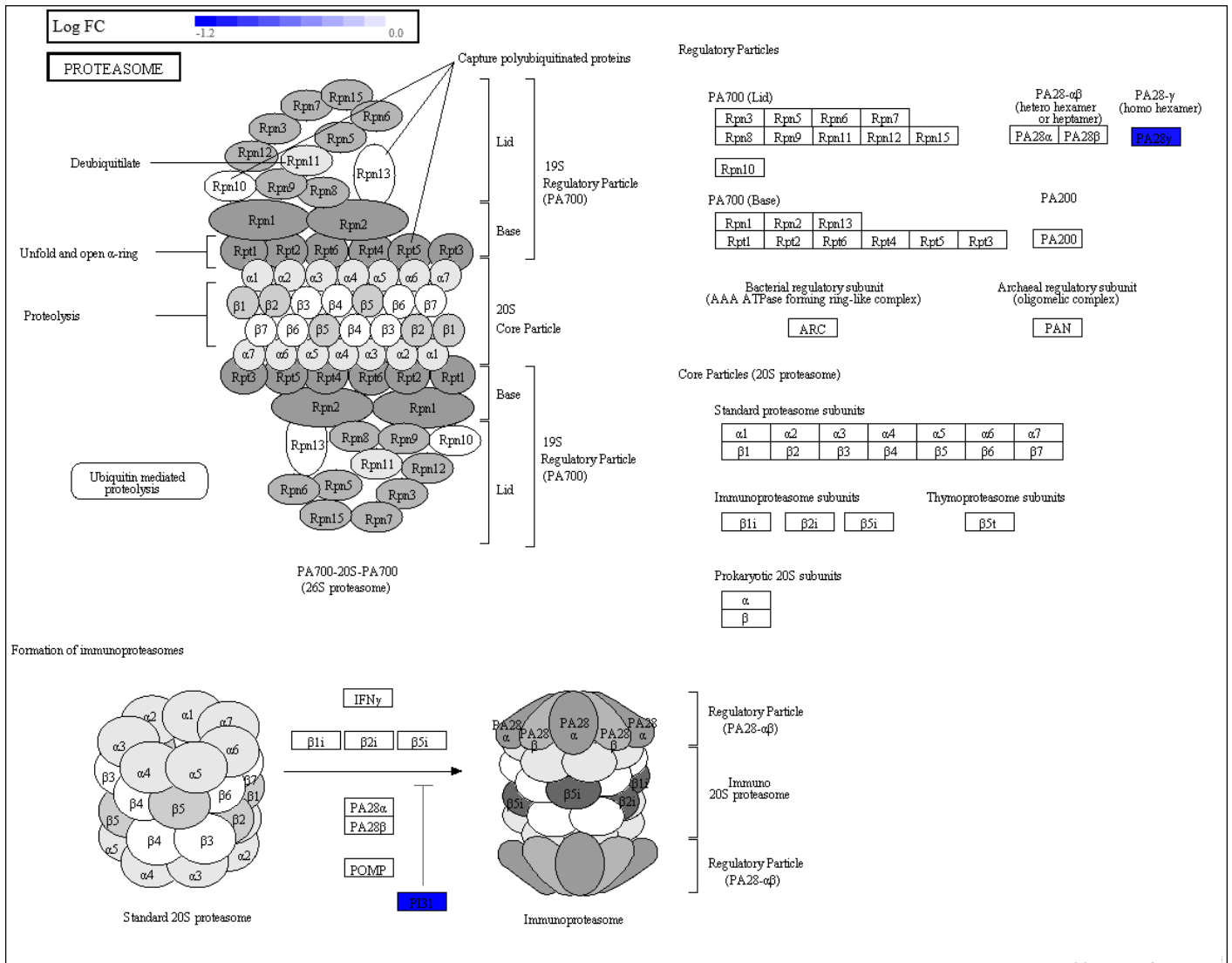

b

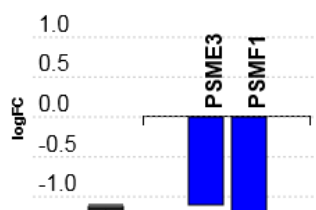

Supplement: Supplementary file 1 [file nutrients-11-02984-s001.zip › Figure S6.pdf]
